# Predicting Inpatient Aggression in Forensic Services Using Remote Monitoring Technology: Qualitative Study of Staff Perspectives

**DOI:** 10.2196/15620

**Published:** 2019-09-19

**Authors:** Ben Greer, Katie Newbery, Matteo Cella, Til Wykes

**Affiliations:** 1 Department of Psychology Institute of Psychiatry, Psychology and Neuroscience King's College London London United Kingdom; 2 South London and Maudsley NHS Foundation Trust London United Kingdom

**Keywords:** telemedicine, remote sensing technology, wearable sensors, aggression, risk assessment

## Abstract

**Background:**

Monitoring risk of imminent aggression in inpatient forensic mental health services could be supported by passive remote monitoring technology, but staff attitudes toward the relevance and likelihood of engagement with this technology are unknown.

**Objective:**

This study aimed to explore staff views, specifically potential benefits and implementation barriers, on using this technology for monitoring risk of inpatient aggression.

**Methods:**

We conducted semistructured focus groups with nurses in an inpatient forensic mental health service. We used thematic analysis with two independent raters to identify themes and subthemes related to staff attitudes toward passive remote monitoring. We subsequently checked with members to ensure the validity of the themes identified by the raters.

**Results:**

From January to March 2019, a total of 25 nurses took part in five focus groups. We identified five main themes, one of which concerned the potential benefits that passive remote monitoring could provide for monitoring risk of aggression. Staff suggested it could provide an early warning of impending aggression and enable support to be provided earlier. The remaining themes concerned implementation barriers, including risks to the users’ physical and mental well-being; data security concerns and potential access by third parties; the negative impact of a constant stream of real-time data on staff workload; and design characteristics and user awareness of the benefits of passive remote monitoring.

**Conclusions:**

Passive remote monitoring technology could support existing methods of monitoring inpatient aggression risk, but multiple barriers to implementation exist. Empirical research is required to investigate whether these potential benefits can be realized, and to identify ways of addressing these barriers to ensure acceptability and user engagement.

## Introduction

### Background

Infrequent structured risk assessments for inpatient aggression cannot detect rapid, momentary changes in individual risk factors, limiting their ability to predict imminent aggression. For example, purportedly changeable (dynamic) risk factors have shown nonsignificant changes over service users’ period of treatment [1]. The limited frequency of these assessments may partially explain why they often show low to moderate predictive accuracy for aggression [2,3]. Acquiring a continuous, real-time measure of how the severity of risk factors changes could provide a more accurate understanding of how these fluctuations relate to the risk of aggressive outcomes [4].

Developments in passive remote monitoring technology (ie, wearable sensors) mean that factors previously associated with aggression cross-sectionally can now be monitored continuously, in real time. For example, many devices monitor indicators of autonomic nervous system activity such as electrodermal activity and heart rate variability, previously associated with aggression in laboratory studies [5-7]. Emerging evidence indicates that passive remote monitoring technology can be used to identify changes in these parameters from 1 to 30 minutes before the observable act of aggression [8,9], and with greater temporal precision than existing risk assessments alone. Monitoring these parameters in real time could identify a reliable psychophysiological signature of when aggression may be more likely, enabling support to be offered before this behavior escalates.

While passive remote monitoring technology has the potential to support risk assessments for aggression [10-13], to our knowledge, the views of frontline staff have not been investigated. Numerous implementation barriers for novel digital health care systems often result in low rates of adoption and adherence [14]. For example, staff have reported a potentially beneficial role for mobile phone–based support for individuals with psychosis, but also highlighted concerns such as infrastructure requirements, data privacy, and the level of digital literacy and skills required of both service users and staff [15,16]. The views of end users, including staff, are therefore critical to understanding the desire and need for digital systems to monitor risk of aggression and the barriers that may be faced.

### Objectives

Inpatient aggression is a barrier to rehabilitation that negatively affects service user and staff well-being [17], and passive remote monitoring technology could facilitate appropriate support and de-escalation. However, not taking a user-centered approach or involving staff in the development and introduction of this technology may mean it is not relevant or acceptable for clinical practice [18]. Therefore, the aim of this study was to explore the attitudes of staff toward passive remote monitoring technology for risk of aggression in inpatient forensic mental health services, with a focus on the potential benefits that this technology could provide and barriers to implementation.

## Methods

### Design

This was an exploratory qualitative study using focus groups following a topic guide. We obtained ethical approval from the Yorkshire & The Humber-Bradford Leeds Proportionate Review Sub-Committee, Jarrow, UK (18/YH/0221) and King’s College London Psychiatry, Nursing and Midwifery Research Ethics Panel, London, UK (LRS-17/18-6715).

### Participants

Participants were staff in a medium-secure forensic mental health service in South London, UK, covering a diverse geographical area including areas of high poverty and urban deprivation. Staff were eligible to participate if their role involved direct contact with service users. Nonclinical staff were not eligible, as the aim of this study was to understand attitudes toward passive remote monitoring technology in a clinical context. We conducted recruitment and analysis concurrently, and recruitment stopped when we achieved data saturation, the point at which focus groups stopped yielding new themes [19].

### Focus Group Topic Guide

The topic guide was based on previous reports [15,16] and included issues related to perceived utility, safety and security, and data connectivity requirements (see Multimedia Appendix 1). The topic guide was informed by consultation with 2 service user–caregiver advisory groups, by a systematic review of the barriers to and facilitators of remote monitoring for health care [20], and by incorporating suggestions from senior management staff in the recruitment site during the setup phase of this study, to ensure that we covered topics relevant to the forensic setting.

### Procedure

We approached ward managers for permission to recruit from their ward, and we conducted 5 focus groups in a private room on the participants’ respective wards during staff handover meetings (2 groups) or at a time convenient for participants (3 groups). Discussions were audio recorded and transcribed verbatim (by BG), with personally identifiable content omitted. Participants were provided £10 (about US $12) in cash after the focus groups in recognition of their time. We conducted member-checking focus groups for the primary themes with the same participants so they could suggest any amendments they felt were appropriate [21]. We informed participants that the study was part of a larger project investigating wearable sensors for monitoring the risk of aggression through physical signals. To provide a context for the discussions, we told participants that the focus groups were the first in a series of studies that aimed to investigate whether real-time monitoring of psychophysiological signals could assist in the earlier detection of an increasing risk of inpatient aggression. We presented 2 remote monitoring devices to illustrate the devices. One device (E4; Empatica Srl, Milan, Italy) is worn around the wrist, and the other (Everion; Biovotion Ltd, Zurich, Switzerland) is worn around the upper arm. Although participants were familiar with commercially available wearable devices, the 2 devices presented were novel to them.

### Thematic Analysis

NVivo 12 software (QSR International) facilitated thematic analysis by 2 independent raters (BG and KN). Both read and reread the transcripts, producing a list of initial codes, and then independently collated the codes into a list of candidate themes and subthemes. Both raters’ initial identification of individual codes and overall themes were compared, resulting in an initial agreement rate of 59% and 72% for individual codes and overall themes, respectively. Where there were discrepancies (eg, 1 rater identifying a code or theme that the other had not), both raters discussed these ratings until they reached a consensus, and themes were revised into their final structure.

## Results

### Participant Demographics

**Figure 1:**
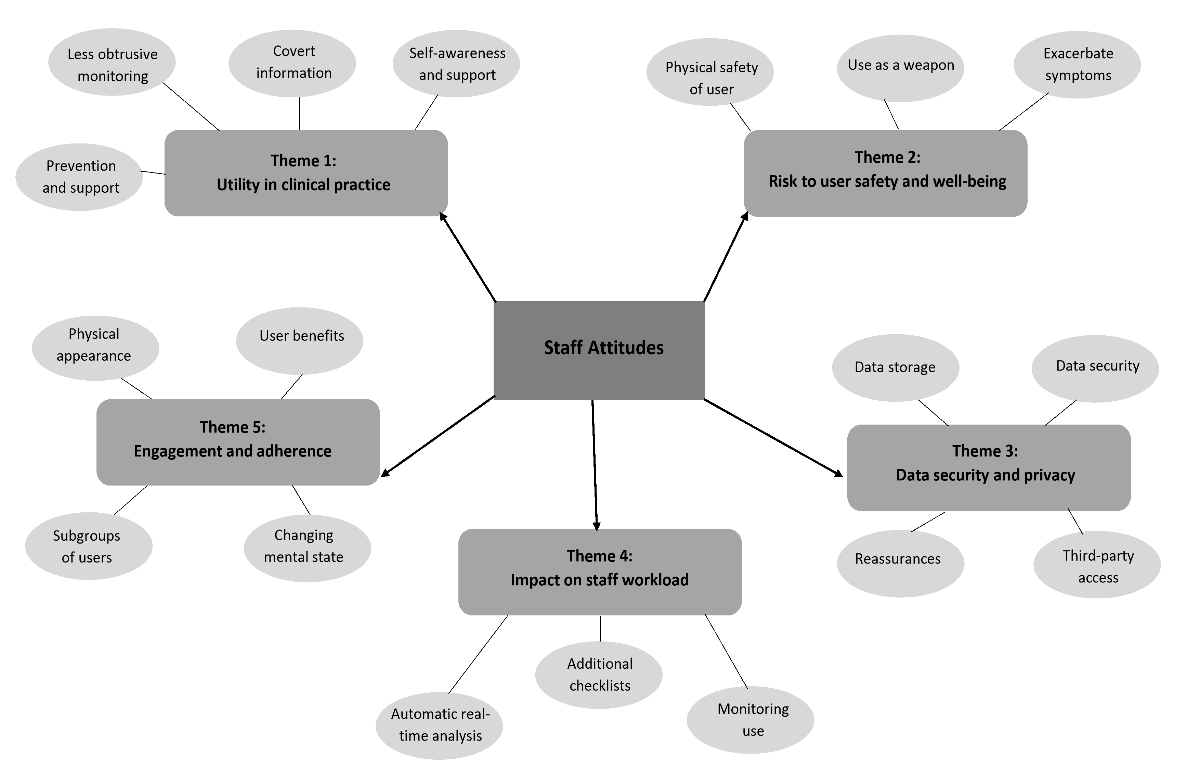
Themes and subthemes identified through thematic analysis.

From January to March 2019, we approached 43 staff, and 25 of them took part in the focus groups. Those who declined did so because of the focus group timing (n=9), or they were required to remain on the ward to maintain minimum staff numbers and carry out clinical duties (n=4); 5 did not specify a reason. A total of 18 participants were also available to take part in the member-checking focus groups. Table 1 presents participants’ demographics. We identified 5 primary themes, which we discuss below in addition to subthemes. Figure 1 provides a visual overview of these themes and subthemes.

**Table 1:** Participants’ demographic characteristics.

Characteristics			Focus group											Total (N=25)			Member-checking groups (n=18)
			1 (n=6)		2 (n=6)		3 (n=4)		4 (n=5)		5 (n=4)						
**Age (years)**																	
	Mean (SD)	37.8 (12.4)		39.5 (11.3)		37 (10.8)		55.4 (7.4)		44.5 (5.1)		42.7 (11.6)			44.4 (12.8)		
	Range	22-54		25-57		25-51		44-64		41-52		22-64			22-64		
**Sex, n**																	
	Female	3		6		3		3		1		16			12		
	Male	3		0		1		2		3		9			6		
**Ethnicity, n**																	
	Black African	4		0		4		4		4		16			15		
	Black Caribbean	1		3		0		1		0		5			1		
	White British	1		3		0		0		0		4			2		
**Job title, n**																	
	Staff nurse	5		6		1		4		4		20			16		
	Student nurse	1		0		2		0		0		3			1		
	Ward manager	0		0		1		1		0		2			1		
**Highest educational attainment, n**																	
	Higher-level qualification (eg, university degree, professional qualification)	5		6		2		5		4		22			17		
	Secondary (A-level equivalent)	1		0		2		0		0		3			1		
Time in post, mean (SD)			5 years, 5 months (3 years, 5 months)		5 years (4 years, 9 months)		4 years (5 years, 8 months)		10 years, 9 months (4 years, 8 months)		4 years, 7 months (2 years, 1 month)		6 years, 6 months (3 years, 5 months)			6 years, 5 months (4 years, 9 months)	

### Theme 1: Utility in Clinical Practice

In every focus group, participants identified numerous ways in which using these devices could augment their practices. One area of discussion related to the devices’ capacity for *prevention and support* to be offered to users:

Yeah it would be helpful, it’s like an early-warning sign so, when you know that they are coming to be anxious, you find a way of intervening before it escalates

Participant 023, nurse

Because sometimes by the time they express it, it means it’s, it’s already you know, so if we can see ahead of time and monitor it, I think it’s good.

Participant 009, nurse

Participants also suggested that these devices could facilitate a *less obtrusive monitoring* approach, enabling assessments without the need for staff to be in physical contact with users:

Because if a patient is wearing this device even if they are in their bedroom, and they’re out of sight of the staff, with the device working you should be able to tell that maybe something has gone wrong...if you just see them physically, they might be in their room they’re anxious, they’re agitated without you seeing them, you won’t be able to tell.

Participant 024, nurse

Reflecting on the seemingly unprovoked nature of some aggressive incidents, participants felt that these devices could provide staff with *covert information* that may not otherwise be expressed by the users or observable by staff:

...because we did not see it we think it’s unprovoked…but with these devices maybe we will know that there’s something happening...before the incident, later maybe attacking somebody or something.

Participant 025, nurse

...not all our patients will be able to say “oh well I feel agitated” or be able to come out and say it, but within themselves all the physical, you know, changes are taking place so I think it’s good, it will help us to see the covert, you know, things that are not outward that the patients cannot express.

Participant 009, nurse

As a result of identifying this covert information, participants felt that this could be used to foster *self-awareness and support* among users, and augment an anger management program offered to users:

...we have taught some of them [service users] who did anger management, but some of them are still not withdrawing when they get to the point of.

Participant 017, ward manager

...[to the] point of anger exactly.

Participant 015, nurse

...this would be a different way of reminding them, maybe this would be a reminder. Because, um, if somebody has done anger management and he knows that by the time he starts breathing heavily, or by the time he feels he’s getting, um, a bit sweaty or agitated and getting wound up...they should disengage. And they have not been doing that because they still don’t have the capacity to do that because they don’t, um, how can I say, they can’t get themselves to...take themselves away from aggression. So, what this would do is to then remind them that this is what they need to do, for some of them who have done anger management.

Participant 017, nurse

### Theme 2: Risk to User Safety and Well-Being

Participants in 4 focus groups discussed the impact of the devices in relation to the *physical safety of the user*. One focus group cited the risk of the device being used as a ligature as a concern, due to the elastic armband of 1 of the devices:

...how far does it stretch, can you put it round your neck?

Participant 008, nurse

Oh yeah, you could I reckon, you could stretch it.

Participant 011, nurse

Well that might be an issue, you know, ligatures.

Participant 008, nurse

The possibility that the devices could be *used as a weapon* was another risk that participants raised, with 1 focus group discussing the potential implications of 1 of the devices having an elastic armband:

...should be something that they cannot use as a weapon, like, there shouldn’t be any metal or something that they can use to self-harm.

Participant 025, nurse

You could use this [referring to device] as a weapon like a slingshot.

Participant 011, nurse

Participants suggested that users’ mental well-being would need to be considered in addition to their physical safety when wearing the devices. Specifically, participants raised concerns that continuous monitoring could *exacerbate symptoms* of paranoia among some users:

They might think that you are monitoring, that you are controlling their mind, controlling their mental state, all of this, so it might make more paranoia.

Participant 003, nurse

When you give this to a paranoid patient they will think you are monitoring them. It will be so difficult to explain it to them to understand it that this is what you’re monitoring....This paranoia could also lead to them not even wearing this.

Participant 015, nurse

### Theme 3: Data Security and Privacy

Across 3 focus groups, participants discussed the measures in place to ensure that data collected by the devices would be kept secure and confidential. Participants wanted to know in advance specific details concerning *data storage*: where the information collected by the devices would be stored and who would have access to it; *data security*: what protections would be in place to keep the data private and confidential; and *access by third parties*: whether data would be shared with other individuals or companies:

...you know I’m gonna need to know, um, what [inaudible] they do, how, even if you say the data is gonna be stored, how secure is the storage, can it be hacked, you know, cos this is like really private, um, information.

Participant 002, nurse

Yeah I think we’d want reassurance, wouldn’t we, that the information we give is protected confidential and it’d be the same for the patients, know how it’s going to be used and...just make sure it’s anonymized the data.

Participant 008, nurse

You know is it gonna be sold to a third-party like we’ve seen with social media now where obviously data protection is like a lot

Participant 002, nurse

Because of these concerns, participants felt that *reassuring users* that their data would be kept secure would be necessary, particularly due to the risk of exacerbating symptoms of paranoia discussed in theme 1:

Just reassuring them that their data will not go anywhere, it’s just for the ward, because some of them will be paranoid.

Participant 025, nurse

One participant made a practical suggestion as to how users could be reassured, drawing parallels with a ward policy whereby staff model appropriate eating during mealtimes. They suggested that staff members wearing the devices themselves could reassure users:

...each time they are eating you need to have 2 staff that will come and model and sit with them...so I’m just thinking that maybe they can try to [inaudible]. If they are wearing it that they see staff wearing it as well, they won’t be thinking about confidentiality, maybe they are trying to take their information or do something else.

Participant 024, nurse

### Theme 4: Impact on Staff Workload

While identifying ways in which these devices could be implemented in clinical practice, participants in 3 focus groups also highlighted that this should not increase staff members’ already high workload. Participants stressed the need for *automatic real-time analysis* of the data collected by the device, to ensure that information can be acted on without additional burden on staff time:

Yeah I mean that’s the only way I can think it’d be useful, without that real-time information, we’re gonna have to take the watch and then upload the data and see what’s going on.

Participant 002, nurse

Yeah [if] it’s automatic, and we don’t have to put in a lot more to get the data and to analyze the data, then it will be good, yeah. But if we have to put in a lot more to measuring and analyzing the data and doing deductions for ourselves, that means additional work to do.

Participant 009, nurse

Participants also questioned whether *additional checklists* would accompany the devices, increasing the level of ongoing input required from staff:

So does this come with a pack or a checklist or something that you’ve got to fill it out every day during the monitoring, or will you really only attend to it when you, it gives you any signals or something that there are any changes? How is it done, I’m just thinking if it’s something that’s supposed to be monitored every now and then and every day it means additional work, isn’t it, you feel, more boxes to tick.

Participant 009, nurse

Responsibility for ensuring that users are wearing the devices was also discussed, with participants feeling that it would fall to staff to spend additional time *monitoring use*, therefore taking time away from their other duties:

Because it’s going to be more [inaudible] on staff now. Now they are wearing it they are gonna say, oh, gonna spend a couple of minutes encouraging them to put it on or go put it on, so it’s going to take valuable time out of your working day, so, it’s going to be time consuming in a way.

Participant 001, nurse

### Theme 5: Engagement and Adherence

All focus groups discussed the numerous factors that may affect the likelihood of users engaging with the devices. The *physical appearance* of the device, including overall size and possible resemblance to a tracking device, was 1 factor:

And you know this one [referring to device] is so conspicuous it looks so much like a tracking device, you know

Participant 009, nurse

...even if they have the reservations about, “oh we don’t want to be monitored” and things like that, if they see something that looks a bit stylish they might be more prone to wear it.

Participant 018, nurse

Participants also suggested that users would be more likely to engage with the devices if there was a clear *benefit to the user*:

If there’s nothing for them they won’t take it.

Participant 017, nurse

One benefit that participants felt would appeal to users was whether wearing the devices would positively affect their leave status:

But then I’m thinking it’s one thing, how is it gonna directly benefit them, like, “what are you gonna tell if I’m a patient, and you wanna give me this I need to know, like...is it gonna make my leave better

Participant 002, nurse

A total of 4 focus groups discussed the impact that *changing mental state* would have on users’ engagement, suggesting that *subgroups of users* may be most likely to engage:

...that would be a problem, getting them to volunteer for it and, um, making sure they understand completely, cos some people are more paranoid on days...than other days so it could be they’re fine for 5 days then the sixth day they’re really paranoid

Participant 008, nurse

The most settled patients there, they will cooperate, some of them are, so most of them that are eager to go out they’ll cooperate, but this ones, like, very paranoid like you said, you will have a tough time.

Participant 001, nurse

## Discussion

### Principal Findings

To our knowledge, this is first study to investigate the attitudes of frontline staff members in inpatient forensic mental health toward passive remote monitoring for risk of aggression. Staff suggested this technology could benefit their assessments, identifying changes in risk factors that would otherwise not be identified. The real-time stream of information provided by these devices could facilitate targeted support before behavior escalated into aggression. However, staff also raised numerous implementation barriers, including the physical safety of the user and security of their personal data, negative impact on staff workload, and engagement barriers.

### Advantages of Passive Remote Monitoring

Participants suggested that the covert information monitored by these devices may account for the seemingly unprovoked nature of some aggressive incidents. Current risk assessments are rated by staff on the basis of observable characteristics (eg, irritability and following instructions [22]); therefore, passive remote monitoring could provide a more complete clinical picture, consistent with previously hypothesized benefits of digital technology for managing aggression [13]. These additional objective data may also circumvent limitations of structured risk assessments such as rater bias [23] and incomplete or inaccurate ratings [24].

Participants suggested that the information provided through passive remote monitoring technology could equip them with prior knowledge of when users may be experiencing difficulties, thereby facilitating appropriate prevention and support. Participants discussed this in relation to staff-initiated de-escalation procedures, but also identified an opportunity to foster self-awareness and support to enable users themselves to de-escalate. This is a novel suggestion for the role of passive remote monitoring in managing aggression, with previous literature typically discussing only how this technology could enable staff to manage risk [13]. Passive remote monitoring could therefore enhance users’ ability to identify and manage their unique risk factors for aggression, consistent with the UK national guideline’s [25] calls for greater emphasis on individual self-regulation [26].

Enabling users to be monitored without the need for in-person observation was considered less obtrusive than current observation practices. Enhanced observations (eg, eyesight and arm’s-length observations) are experienced negatively by both service users and staff [27]; therefore, passive remote monitoring may enable monitoring with fewer physical restrictions. This would need to be balanced with the accuracy and range of clinical observations that can be made with passive remote monitoring alone. For example, peer interactions and negative attitudes are relevant risk factors for aggression [28] but cannot be assessed through actigraphy or biosensors alone, highlighting the need for multiple sources of observation data. Overreliance on technology could also limit the opportunity for physical service user–staff interactions and dialogue, an integral component of therapeutic relationships [29]. As mentioned in 1 focus group, however, providing staff with feedback on changes in psychophysiological parameters of individuals in their care could also facilitate dialogue and staff–service user interaction (“I could see that it could be useful, because, um, it could just be a point of engagement for staff” [Participant 017, nurse]).

The suggestion that passive remote monitoring would be suitable only for subgroups of users is consistent with a previous evaluation of a passive remote monitoring system (a global positioning system [GPS] tracker) in a forensic mental health service [30]. The system in that study was primarily used for subgroups at the early stages of their leave period or during specific periods of transition. Participants felt that these subgroups would be based on users’ current mental state and paranoid ideation. Passive remote monitoring may not be suitable for everyone and reflects the need for a personalized approach, which balances the potential benefits to the user (eg, improved understanding of changes in risk state) and challenges (eg, difficulties with engagement).

While highlighting the potential benefits of passive remote monitoring technology, no participants suggested that technology should replace the practice of staff-completed risk assessments. This is consistent with previous research reporting universal agreement among staff that digital health care technologies should be an adjunct to traditional care rather than a replacement, as replacement could be detrimental to user well-being and therapeutic relationships [16]. Future use of passive remote monitoring should therefore be considered as a component of a blended approach that complements, but does not wholly replace, staff-completed structured risk assessments.

The issues discussed above are hypothesized benefits, and while there is potential for passive remote monitoring technology to support risk management for aggression, this needs to be supported by high-quality empirical evidence. Key issues that need to be addressed include the feasibility and acceptability of this technology for end users, whether a reliable psychophysiological signature for aggression exists, and the accuracy of the data provided by this technology, including the ability to correctly identify changes related to aggression and to rule out those that are unrelated.

### Implementation Barriers

Participants identified numerous issues that are likely to affect successful implementation of passive remote monitoring systems. The physical safety concerns raised by participants, relating to ligature risk and use as a weapon, appeared to be linked to a specific design characteristic (elastic armband) of a device presented during the focus groups. This highlights the importance of considering the physical design of passive remote monitoring devices intended for use in inpatient services, where physical safety and risk of self-harm are management priorities. Concerns were raised that continuous passive remote monitoring may exacerbate symptoms of paranoia; therefore, establishing trust with users beforehand is likely to be integral to successful implementation. While previous research of passive remote monitoring in the community indicates the acceptability of passive remote monitoring for individuals with psychosis [31-33], it may be a pertinent issue for those involuntarily admitted to inpatient services and experiencing loss of control and restrictive practices [34].

Consistent with previous research among staff [35,36] and service users [37], participants expressed data privacy concerns. Rather than voicing a general unspecified concern, participants specified 3 areas of assurance they would require to be comfortable with passive remote monitoring, relating to data storage, security, and accessibility by third parties. Addressing these concerns in the long term will require digital health companies to be transparent about the procedures in place for handling user data, and to ensure that users have access to this information. In the shorter term, participants reported that staff could play an important role in providing reassurance to users. The suggestion that staff could lead by example by trialing the devices themselves reflects the role of staff as positive role models in inpatient services [38] and is a practical approach to alleviating user concerns.

Participants expressed concern that incorporating passive remote monitoring into their working practice might negatively affect their existing workload. Like Bucci et al [16], participants were concerned with the potential burden of handling and analyzing large volumes of real-time data, and emphasized that these devices need to be complemented by automatic real-time analysis. While this would address the process of analyzing the raw data into an actionable format, it is possible that a constant stream of processed data could still prove overwhelming. It will therefore be important to establish an appropriate format for presenting the data, balancing the frequency of data, level of detail, and staff capacity to act on this information. Participants also highlighted practical considerations such as the introduction of additional checklists with passive remote monitoring and questioned where responsibility for monitoring use would lie. Future use of passive remote monitoring devices will therefore need to balance potential clinical benefits with practical implementation issues, to ensure that they support and do not hinder clinical care. Machine learning algorithms to process data in real time and present it in a user-friendly and actionable format would be an option, and participants discussed presenting the data as a visual display in the ward’s central nursing office. Embedding a dedicated technology specialist within the clinical team could also be considered [39]. While not raised in the focus groups, there are likely to be numerous practical implementation issues to using remote monitoring technology, such as the financial cost of acquiring and maintaining the technology, and any ongoing training requirements for staff.

Achieving the hypothesized benefits of passive remote monitoring devices requires adequate levels of user engagement. The influence of a device’s physical appearance on engagement highlights the importance of involving users in the choice of devices, as individual preferences may vary. User involvement in the design of these devices could also be an effective way of ensuring that they are considered acceptable. Engaging in passive remote monitoring also needs to have discernible benefits to the user, which in the context of managing risk of aggression could include less restrictive management practices. Communicating these potential benefits could therefore have a positive effect on engagement. User engagement will also entail staff enthusiasm to work with novel digital technologies. Barriers to organizational change within mental health services include poor job satisfaction among staff, burnout, and lower levels of experience [40,41], and these may also be barriers to the successful introduction of passive remote monitoring technology.

### Strengths and Limitations

This study took place in a hospital where passive remote monitoring technology, GPS tracking devices, has been in use for several years to monitor leave [30,42]. Participants’ responses may have been influenced by this prior experience and so may not necessarily reflect the views of those unfamiliar with these systems. However, some familiarity might also have facilitated discussion, with the issues and recommendations raised reflecting participants’ applied experience of passive remote monitoring. Because this is a medium-secure forensic mental health service, the themes identified in this study may not necessarily generalize to forensic services of higher levels of security (eg, where more stringent policies for patient access to digital equipment exist), or to non–forensic mental health services, where inpatient aggression also occurs.

Senior nursing and managerial staff, whose views might have provided greater organizational context, were present in smaller numbers and so we did not successfully capture their views. Including only members of the nursing staff may have limited the emergence of new themes. For example, the technical infrastructure requirements for remote monitoring technology could be clarified by seeking the views of support service staff (eg, technicians and engineers). The time for focus groups was restricted, and this might also have limited the emergence of new themes. However, the replication of themes across the different groups makes this unlikely.

### Conclusion

Passive remote monitoring technology offers potential benefits to monitoring risk of aggression in inpatient forensic mental health services. However, novel digital systems in mental health have generated substantial hype [43], and these potential benefits have yet to be realized through high-quality empirical research. Future research should therefore investigate whether passive remote monitoring is able to achieve the benefits suggested by staff and reliably identify increased risk of aggression. Specifically, future research should investigate the areas of concern identified by staff in this study to determine acceptability and feasibility of passive remote monitoring, such as whether subgroups based on diagnosis are more likely to engage, and the impact of real-time monitoring on staff workload. Determining whether a reliable psychophysiological signature of imminent aggression exists is also critical, and robust methods for analyzing these data, such as machine learning, will need to be developed and evaluated to make this determination.

## Appendix

Multimedia Appendix 1 Focus group topic guide.

## Abbreviations

GPS: global positioning system
