# Supplementary material for: Predicting Inpatient Aggression in Forensic Services Using Remote Monitoring Technology: Qualitative Study of Staff Perspectives
Source: J Med Internet Res. 2019 Sep 19;21(9):e15620. doi: 10.2196/15620 (PMC6754691; doi:10.2196/15620)
Supplement: Multimedia Appendix 1 [file jmir_v21i9e15620_app1.pdf]

Supplementary Appendix 1
Focus Group Topic Guide

- Can you describe what wearable technology is?
  Prompts:
    - *Do you know what can they be used for?*
    - *Can you give any examples of devices you know of/have used?*

- Has wearable technology been used here? If so can you describe its use?

- Could you see any potential barriers to using this kind of technology on the wards?
  Prompts:
    - *Safety (e.g. self-harm, weapon), security, and privacy?*
    - *Data connectivity issues (e.g. transferring the data from device to computer server)?*
    - *Time/practicality (e.g. training in device use, analysing the data)?*
    - *Fitting into current routines/work practices*
    - *Health-related barriers*
    - *Perceived usefulness of this technology*

- Previous researchers have suggested wearables could be used to monitor physical 'warning-signs' of aggression (e.g. heart rate, electrodermal activity). What are your thoughts on this?
  Prompts:
    - *Would this information be useful in your day-to-day work?*
    - *Would you have any concerns about doing this (e.g. privacy)?*
    - *Do you think service users would have concerns about doing this?*

- Is there anything else you think wearables could be used for within your service?
  Prompts:
    - *Do you think they could have any role at all? If not, why?*
